# Resident dashboards: helping your clinical competency committee visualize trainees’ key performance indicators

**DOI:** 10.3402/meo.v21.29838

**Published:** 2016-03-31

**Authors:** Karen A. Friedman, John Raimo, Kelly Spielmann, Saima Chaudhry

**Affiliations:** 1 Department of Medicine, Hofstra Northwell School of Medicine, Hempstead, NY, USA; 2 Internal Medicine Residency Program, Hofstra Northwell School of Medicine, Hempstead, NY, USA

**Keywords:** clinical competency committee, Next Accreditation System, reportable milestones

## Abstract

**Introduction:**

Under the Next Accreditation System, programs need to find ways to collect and assess meaningful reportable information on its residents to assist the program director regarding resident milestone progression. This paper discusses the process that one large Internal Medicine Residency Program used to provide both quantitative and qualitative data to its clinical competency committee (CCC) through the creation of a resident dashboard.

**Methods:**

Program leadership at a large university-based program developed four new end of rotation evaluations based on the American Board of Internal Medicine (ABIM) and Accreditation Council of Graduated Medical Education's (ACGME) 22 reportable milestones. A resident dashboard was then created to pull together both milestone- and non-milestone-based quantitative data and qualitative data compiled from faculty, nurses, peers, staff, and patients.

**Results:**

Dashboards were distributed to the members of the CCC in preparation for the semiannual CCC meeting. CCC members adjudicated quantitative and qualitative data to present their cohort of residents at the CCC meeting. Based on the committee's response, evaluation scores remained the same or were adjusted. Final milestone scores were then entered into the accreditation data system (ADS) on the ACGME website.

**Conclusions:**

The process of resident assessment is complex and should comprise both quantitative and qualitative data. The dashboard is a valuable tool for program leadership to use both when evaluating house staff on a semiannual basis at the CCC and to the resident in person.

As part of the reporting process in the Next Accreditation System (NAS), residency training programs must develop a robust clinical competency committee (CCC) (1) that monitors resident progress against predefined milestones using a combination of assessment methods (2, 3). The members of the CCC are charged with making recommendations to the program director (PD) regarding resident progress. Programs will need to find creative and efficient ways, by which the CCC can aggregate, analyze, display, and report educational outcomes for each resident (4).

The most recent report of the Institute of Medicine (IOM) on graduate medical education (GME) calls for more transparency in the achievement of GME goals (5). This is a very complex process that can be aided by the use of dashboards in residency programs. Dashboards were originally developed in the business world to enable easy access to multiple sources of data. These dashboards are often in a visual, concise, and usable format (6). Many hospitals and health systems already measure physician performance, presenting data to the practitioner, via either a report card or dashboard (7). Physician report cards currently can report on both clinical-outcome-based measures of care quality as well as non-clinical-outcome-based measures of care quality such as patient hospital experience data (8). Similarly, resident dashboards help hold individuals accountable for their success by supplying them with dashboards of their milestone achievements. Resident dashboards can potentially enact multiple functions simultaneously. One is to allow the residents to view the progression of their individual milestone achievements and focus on areas in need of improvement. Another is providing the program with an overall view of the progression of each resident thereby identifying a struggling resident earlier. Finally, dashboards can give an overall progress of the quality of the program to see if program goals for trainees are being met and if trainees are being trained appropriately.

In this paper, we aim to report the process by which one large Internal Medicine Residency Program created a dashboard to supply the CCC with qualitative and aggregate quantitative milestone data on each resident.

## Methods

The Hofstra Northwell School of Medicine Internal Medicine Residency Program is a large, university-based program consisting of 131 residents rotating through two tertiary hospitals and two ambulatory clinics. The program has one PD, two site directors, and five associate program directors (APDs). House staff are evaluated by the attending that they worked with at the end of all of their rotations using an electronic-scale-based rating form through a web-based residency management suite (RMS).

**Fig. 1:**
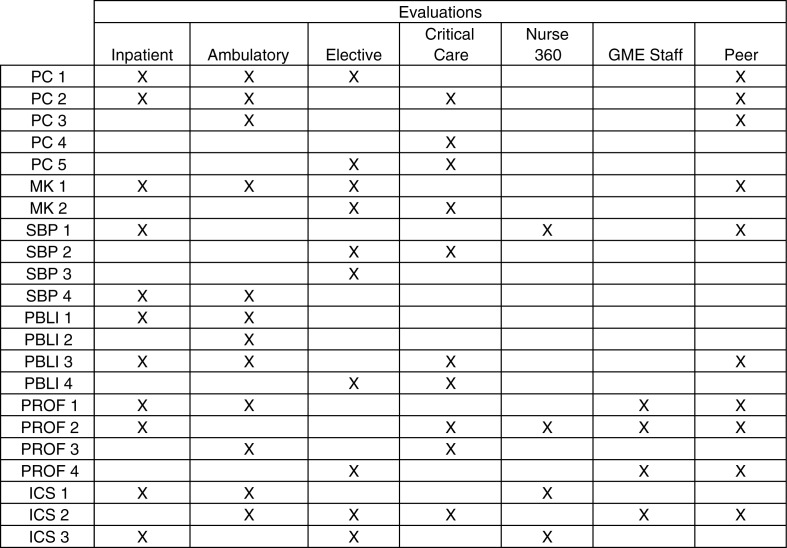
Milestone distribution among resident evaluations.

The program created four different rating-based end of rotation evaluation forms that are aligned with the 22 reportable milestones developed by the American Board of Internal Medicine (ABIM) and Accreditation Council of Graduate Medical Education (ACGME) (9). Faculty leadership met and determined the appropriate milestones for each rotation. The new evaluations were structured such that the sum total of all 22 reportable milestones was represented at least once within the four evaluations. The inpatient evaluation encompasses all floor rotations, medical admitting resident rotations, and night float rotations. Ambulatory evaluations encompass ambulatory clinic rotations, private practice rotations, and geriatrics rotations. Critical care evaluations include medical intensive care unit (MICU) rotations, coronary care unit (CCU) rotations, and emergency room rotations. The elective evaluations encompass all of the rotations that our residents complete in each of the medical subspecialties (cardiology, endocrinology, gastroenterology, infectious disease, nephrology, palliative care, pulmonology, rheumatology, etc.). The milestones attached to each of the four evaluations can be found in Fig. 1. All evaluations also have blank text boxes to encourage written feedback. The resident peer-to-peer evaluations, 360° evaluations completed by the nursing staff, and the GME staff evaluations were also modified in order to evaluate the residents based on the reportable milestones (see Fig. 1).

**Fig. 2:**
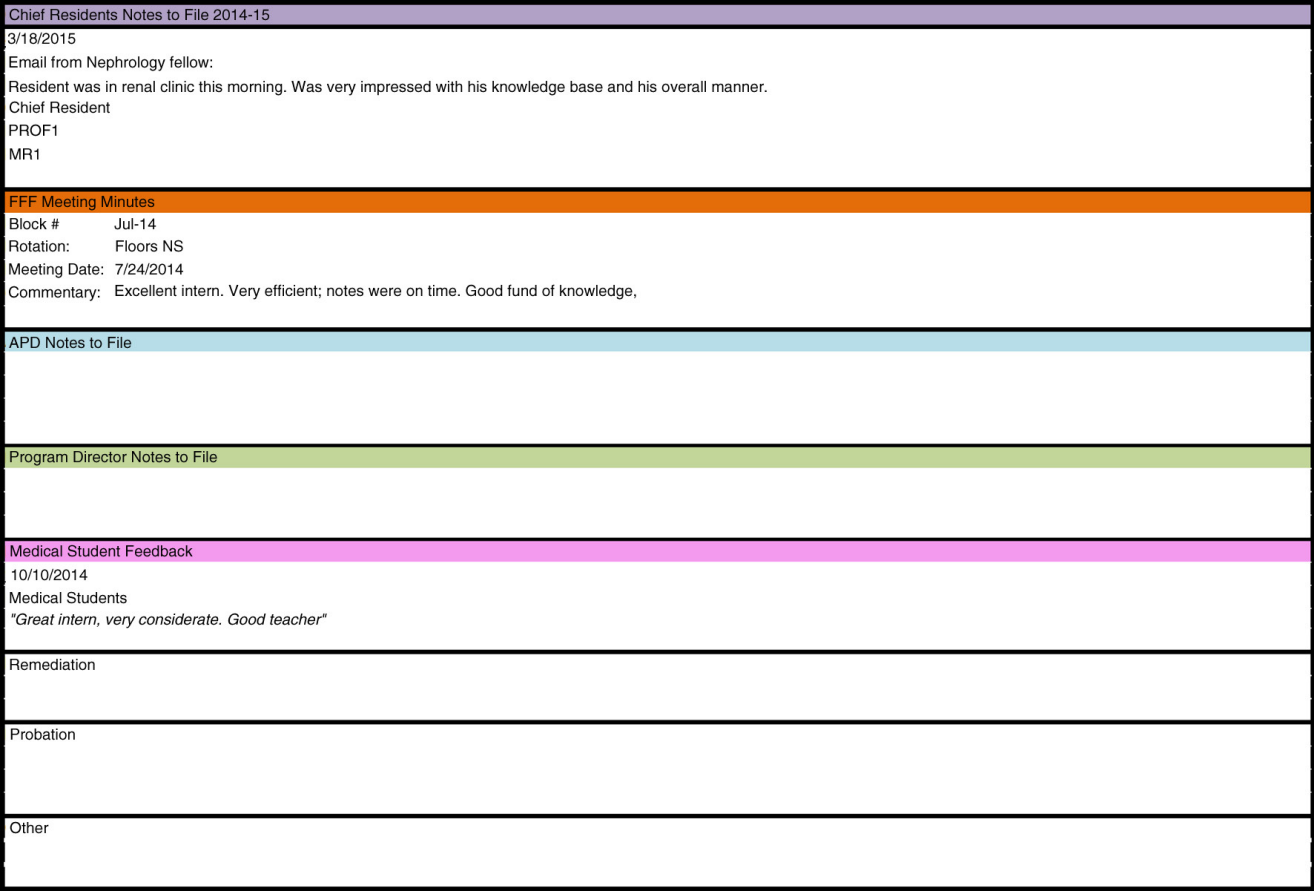
Qualitative section of the milestones dashboard.

Once the creation of new evaluations was completed, a dashboard was developed that would compile the data for each resident from all of the assessment tools in one spot. The dashboard was created in Microsoft Excel with each resident having their own worksheet in the Excel document. A quantitative summary score for each resident's 22 milestones was calculated using a weighted average, with faculty evaluations at the end of the rotation rating forms holding twice the weight of nursing, peer, or staff evaluations. The dashboard also includes non-milestone quantitative data: in-training exam scores, duty hour logging compliance, RMS evaluation compliance, Mini-CEX compliance, and the resident's rank compared with his/her post graduate year level (1–10 scale). A resident's rank is obtained through a separate question that is administered to the faculty in their RMS evaluations. To get the global scope of a resident's performance, the dashboard includes a separate section that contains qualitative data. The qualitative data include notes of incidents, accolades, and unsolicited verbal feedback provided by faculty, nurses, peers, staff, or patients to program leadership. These comments can come in the form of emails, letters, or direct face-to-face communication. These data are located at the bottom of the dashboard and are separated into the following sections: chief residents’ notes to file, faculty feedback forum (FFF) meeting minutes, APD notes to file, PD notes to file, medical student feedback, remediation, probation, and others. FFF is a monthly meeting where faculty meets with the program site directors and gives verbal feedback on the house staff. Minutes are recorded and comments are entered into the dashboard as need by GME staff. Any notes received from the PD, APDs, chief residents, and the various other program staff are recorded in this section of the dashboard (Fig. 2).

**Fig. 3:**
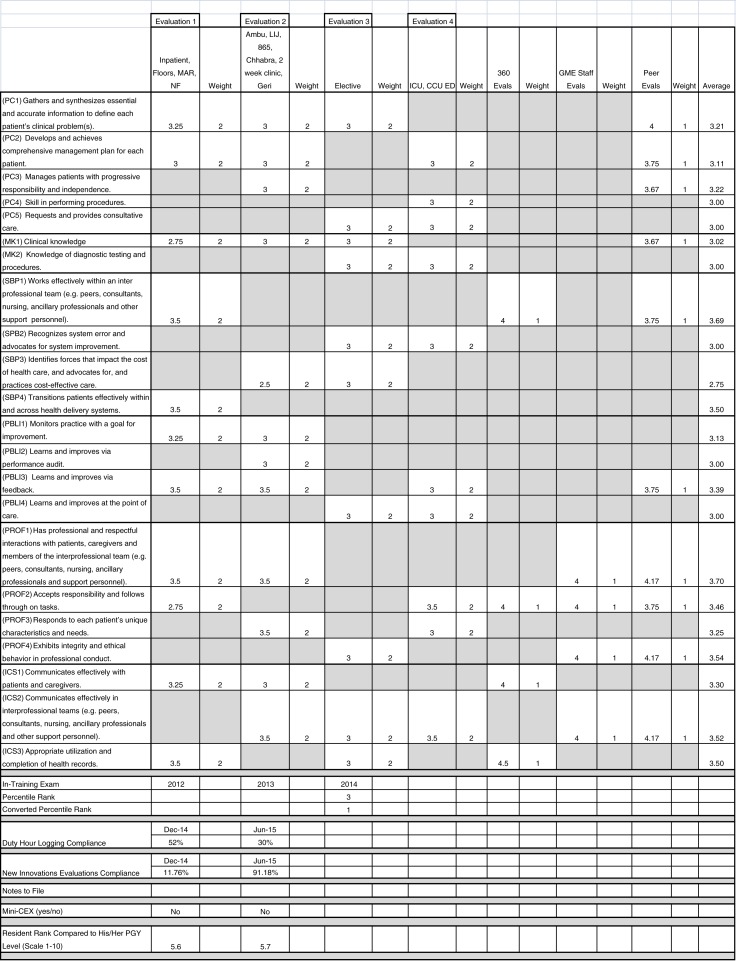
Sample milestone dashboard.

Figure 3 depicts an image of the dashboard for one resident. The 22 reportable milestones are listed as separate rows. Each column represents one of the evaluations, followed by a column containing the weight of that milestone and evaluation. Milestones and evaluation data intersect at multiple points across the dashboard. Cells that are blacked out indicate that the particular milestone was not assessed by that evaluation. Faculty evaluations were given a weight of 2, while peer evaluations, 360° evaluations, and GME staff evaluations received a weight of 1. The last column of the dashboard is a calculated weighted average of each milestone based on the aggregate evaluation data.

## Results

The first trial of the dashboard occurred in December 2014. Evaluation summary reports and compliance reports for each of our 131 residents were downloaded from the RMS. The scores from these reports were manually entered into each of the resident's Excel dashboards. One GME staff was tasked with collating all the qualitative data from each resident's RMS file and pasting it onto the corresponding resident's dashboard. The required time was approximately 5 min for GME staff members to enter each of the resident's qualitative and quantitative data sets onto the dashboards. The process took approximately 11 uninterrupted hours in total to enter all information into the dashboard [131 residents×5 min=655 min (10.91 h)].

After the dashboard was completed for all of the program's residents, they were disseminated to the APDs and PDs that constitute the CCC. Each of these members received an assigned set of residents to review as well as a blank copy of the ACGME milestones evaluation that is to be entered into the accreditation data system (ADS) on the ACGME website. Using the dashboard information, each member completed the ACGME milestones evaluations on their set of residents. Each CCC member spent approximately 30 min per resident evaluating dashboard information prior to the CCC meeting. The suggestion for final milestone scores to the CCC was determined at the discretion of the APD/PD whose job it was to adjust the final assessment to be entered into ADS based on the quantitative and qualitative data present in the dashboard. At the CCC meeting, it was the responsibility of the APD/PD to present their set of residents to the committee at large. Based on the committee's response, the evaluation scores will remain unchanged or will be adjusted. On site, as the CCC meeting was taking place, a member of the GME staff entered the final milestones scores into the ACGME website. The CCC meeting took approximately 5 h to review all 131 residents. A disproportionate amount of time was spent on residents whose scores were being adjusted based on combining of quantitative and qualitative data. Many residents were progressing without issues and did not require extensive discussion.

## Discussion

Resident assessment is a complex task that should include a comprehensive review of aggregate rating-based scores (most frequently determined through rating forms based on end of rotation scale) and qualitative data. In the NAS, this reporting is done biannually to the subspecialty boards via the reportable milestones. The standardization of the process was the key to making our data pull and report out of the CCC run smoothly and accurately.

While the first official data submission was due for our program in June of 2015, we piloted our new database in December of 2014. Expertise in data management and residency software management systems is a key component for success (10). By the time we started the second round of data extraction in May of 2015, our RMS had added features that lessened the time for data pull.

The creation of our database provided a standardized format to review quantitative and qualitative multisource data in a comprehensive fashion. Each APD in preparation for the CCC was able to review the quantitative data of all 22 reportable milestones and combine them with qualitative information collected throughout the prior 6 months. It is not unusual for programs to find grade creep on the quantitative evaluations done by faculty. Faculty members are often not willing to grade on the lower end of the scale (11). This format allowed the APDs to shift scores when combining the data. For example, if a resident scored well quantitatively on Professionalism 1 (PROF 1) (has professional and respectful interactions with patients, caregivers, and members of the interprofessional team) but the notes to file (qualitative data) reveals professionalism issues with the chief residents or nurses, the final reported score could be lowered (9). At the other end of the spectrum, residents who scored average for Systems Based Practice 1 (SBP 1) (works effectively within an interprofessional team) but has letters in their file with praise from nurses and case managers about their collegiality can have their final reported score raised (9). The key to success is the standardized review by each APD of the data and a final agreed upon score by the CCC as a whole.

The dashboard is now a valuable tool to use when sitting down with house staff for their semiannual evaluations. Not only can they view the composite scores of all of their evaluations but there is easy access to qualitative data for them to view that does not reside in the RMS. The process becomes transparent to the house staff as the APD is able to explain why scores were moved in certain directions. Review of the data in this format also brought to light more residents who needed to be placed on remediation. Often when the quantitative data for particular milestones were borderline, the qualitative data dragged the score further down, leading to raising of red flags and initiation of discussions regarding remediation.

Like all dashboards, educational dashboards are not without their limitations. Their main function is to display data and inform residents of their progress. At this juncture, we do not expect the information presented to change resident behavior. The goal is to make residents more aware of the components that factor into their overall evaluation. Compiling our data in this fashion in fact did not save us any time. Prior to the creation of the dashboard, our program was not gathering all the data in a standardized, comprehensive fashion such that all of the data existed in one place. For us, the real value of the dashboard lies in its comprehensiveness.

Going forward, one could transform the dashboard into a more fluid model as opposed to the static model that currently exists in Microsoft Excel. Leveraging the technology that already exists at the institutional level could provide residents and the CCC with dashboards that can trend results and be more visually appealing. In the future, the dashboard could be a residency Global Positioning System (GPS) that provides the user a quick glance at information about where they currently are and where they need to be. Just as dashboards provide physicians with outcomes on their quality metrics, residency dashboards could provide GME programs with metrics on educational outcomes.
